# Pattern recognition receptor CD14 gene polymorphisms in alcohol use disorder patients and its Influence on liver disease susceptibility

**DOI:** 10.3389/fimmu.2022.975027

**Published:** 2022-09-27

**Authors:** Neelanjana Roy, Neeti Nadda, Hem Kumar, Chandreswar Prasad, Jyotish Kumar Jha, Hem Chandra Pandey, Perumal Vanamail, Anoop Saraya, Yatan Pal Singh Balhara, Shalimar, Baibaswata Nayak

**Affiliations:** ^1^ Department of Gastroenterology and Human Nutrition, All India Institute of Medical Sciences, New Delhi, India; ^2^ Blood Transfusion Medicine, All India Institute of Medical Sciences, New Delhi, India; ^3^ Biostatistics in the Department of Obstetrics and Gynecology, All India Institute of Medical Sciences, New Delhi, India; ^4^ Department of Psychiatry and National Drug Dependence Treatment Centre, All India Institute of Medical Sciences, New Delhi, India

**Keywords:** CD14, SNP, inflammation, promoter, alcohol, liver disease, cytokine, genetic association

## Abstract

**Background:**

Alcohol use disorders (AUDs) leading to liver disease is major concern over other spectrum of disorder. Excessive alcohol consumption resulting in leaky gut syndrome is attributed to alcohol-induced liver injury through portal translocation of bacterial endotoxin. Susceptibility to alcoholic liver disease (ALD) in AUD patients could be dependent upon genes responsible for inflammation and alcohol metabolism. The pattern recognition receptor CD14 gene is a major player in endotoxin-mediated inflammation and susceptibility to ALD. This study investigated the genetic association of CD14 polymorphisms and other mechanisms relevant to altered inflammatory responses leading to ALD.

**Methods:**

Patients with alcohol use disorder with ALD (n **=** 128) and without liver disease (ALC, n = 184) and controls without alcohol use disorder (NALC, n = 152) from North India were enrolled. The CD4 gene polymorphisms in the North Indian population were evaluated by RFLP and sequencing. Secretory CD14 (sCD14), LBP, TLR4, MD2, TNFα, IL1b, IFNγ, IL6, IL10, and IL4 levels in serum were measured by ELISA among groups. The influence of polymorphisms on CD14 gene promoter activity and circulatory bacterial DNA level was determined.

**Results:**

The CD14 gene promoter and exonic region SNPs were found to be monomorphic, except for SNP rs2569190 for the North Indian population. The genetic association of SNP rs2569190(C/T) with the risk of developing ALD was found significant for TT genotype [OR_TT_, 95% CI = 2.19, 1.16–4.13 for ALD vs. ALC and OR, 2.09, 1.18–3.72 for ALD vs. NALC]. An increased sCD14 level was observed in AUD patients compared to NALC control. Increased levels of LBP, TLR4, TNFα, IL1β, IFNγ, and IL6 and reduced levels of MD2, IL10, and IL4 were observed among the ALD patients compared to the other two control groups. Elevated levels of pro-inflammatory and reduced levels of anti-inflammatory cytokines were observed in the risk genotype TT groups of ALD patients and the ALC group compared to NALC. Promoter activity was observed in the intronic region flanking SNPs and risk genotype can influence reporter activity, indicating CD14 gene expression.

**Conclusion:**

Enhanced CD14 expression associated with inflammatory responses increases susceptibility to ALD in the TT genotype of AUD patients.

## 1 Introduction

Alcohol use is a major concern for both present and future health. Alcohol as risk exposure is increasing at a rate of more than 0.5% per year (1). Alcohol use disorders (AUDs) including alcohol harmful use, dependence and alcohol induced depressive disorders are widespread in the general population and constitute a huge public health burden worldwide (2). Alcohol use is the top leading risk factor attributable to disability-adjusted life-years (DALYs) among age groups of 25–49 years. Alcohol abuse attributable to mortality accounted for 2.07 million (1.79–2.37) deaths in males and 0.374 million (0.298–0.461) deaths in females as reported in the Global Burden of Diseases, Injuries, and Risk Factors (GBD) Study, 2019 (1). Out of all forms of global injury burdens, cirrhosis ranks 9th and alcohol is considered the third modifiable cause of it, following chronic hepatitis B and C (CHB and CHC) infection (1, 3). Alcohol use was responsible for 5.9% of all deaths and 5.1% of the global burden of disease and injury as measured in DALYs in the year 2012 (4). Chronic alcohol consumption in larger amounts (>80 gm/day) for a more extended period causes liver injury-inducing alcoholic liver disease (ALD). The spectrum of ALD includes stages of alcoholic fatty liver; chronic hepatitis progressing through fibrosis to cirrhosis; hepatocellular carcinoma (HCC) and death. Alcohol will soon emerge as the leading cause of cirrhosis over CHB and CHC due to the availability of vaccines and potent drugs against hepatitis B and C viruses (5). Acute excessive alcohol consumption or binge drinking in underlying chronic liver disease (CLD) or hepatitis A or E virus infections in alcohol-CLD also induces acute-on-chronic liver failure (ACLF). In our recent study, severe presentations of ACLF with higher incidences of organ failures and mortality were observed in patients with alcohol as an acute hepatic insult than hepatitis virus etiologies (6).

ALD severity predominantly depends upon the alcohol dose, duration and drinking patterns. Additional risk factors for progression of hepatic injury beyond fatty liver or severity of ALD are not well defined. ALD severity is significantly predicted by metabolic and genetic risk factors alone (7). Other associated risk factors such as gender, obesity, iron overload, and concurrent hepatitis virus infections also modulate ALD severity (8–10). Alcohol metabolisms differ in individuals due to genetic variations, which may put some people at a higher or lower risk of ALD. Several population-based case-control studies have shown an association of ALD with several single nucleotide polymorphisms (SNPs) of genes related to alcohol and lipid metabolism or pro-inflammatory (11) and anti-inflammatory (12, 13).

Alcohol aggravates inflammation by stimulating the production of cytokines, acetaldehyde toxicity, lipid peroxidation, and oxidative stress. Chronic consumption of alcohol results in bacterial overgrowth, leaky gut, increased circulation of gut-derived lipopolysaccharides (LPS), and decreased phagocytic activity (14). Gut-derived LPS mainly mediates inflammation in ALD with the pattern-recognition receptor (PRR) in the liver, activating signaling pathways for cytokine production (15, 16). CD14 (Cluster of Differentiation 14) is a key PRR for several microbial products, including LPS, and its expression was found to be increased following chronic alcohol ingestion. CD14 interacts with LPS by forming a complex with LPS-binding protein (LBP), Toll-like receptor-4 (TLR-4), and MD2 to initiate production of pro-inflammatory cytokines such as tumor necrosis factor α (TNFα) and interleukin (IL)-1β, as well as diminish the synthesis of anti-inflammatory cytokines such as IL-10 and IL-4 (17, 18). CD14 is normally expressed on neutrophils, monocytes/macrophages, and fibroblasts (19). Chronic alcohol insult results in continuous endotoxin/LPS mediated CD14 activation and proinflammatory cytokine production, ensuing chronic inflammation and sustained hepatocellular damage (20).

CD14 polymorphisms have been found to be related to the development and progression of advanced ALD (21, 22). These polymorphisms are recognized to influence CD14 expression, leading to an enhanced inflammatory response (23, 24). Genetic variations in CD14 polymorphisms may have a role in determining the susceptibility or protection to the severity of ALD, for which the current study was undertaken for Indian populations. A case–control genetic association study of ALD patients versus patients with alcohol use disorder without liver disease and healthy controls was undertaken. Novel genetic variations in CD14 exons and their association with severe ALD along with functional relevance effecting the inflammatory signaling pathway were studied.

## 2 Methods

### 2.1 Study population and design

Four hundred sixty-four consecutive subjects (n = 464) were finally enrolled after exclusion for this association study to identify susceptible polymorphic genetic markers for developing ALD. This case–control association study was designed to determine the risk allele and genotype of CD14 SNPs and their relevant mechanisms associated with severe ALD compared to alcoholic control (ALC) and healthy non-alcoholic controls (NALC). The study was approved by the Institute ethics review committee (No: IESC/T-440/26.08.15, RT-7/2015).

#### 2.1.1 Diagnostic criteria for ALD, assessment of alcohol use disorder, and amount of alcohol consumption

Alcohol use disorders including alcohol harmful use (ICD10, F10.1), dependance (ICD10, F10.2) and alcoholic liver disease (K70.0-K70.4/9) were determined as per as per WHO International Classification of Diseases (ICD) criteria. Alcohol consumption of ≥80 gm per day is considered excessive use. Alcohol volume in ml to gram conversion was carried out by multiplying factor 0.8 according to alcohol density (~0.8 gm/ml). The alcohol concentration in the beverages was determined on the basis of the percentage of alcohol such as whiskey, rum, gin, scotch, and desi liquor (40%), wine and local fermented beverages (5%–15%), and beer (5%–10%). The modified CAGE (Cut down, Annoyed, Guilty and Eye-opener) and AUDIT (Alcohol Use Disorders Identification Test) questionnaires were used for screening and estimation of the level of problematic pattern of alcohol use. Alcoholic liver disease, including fatty liver, hepatitis, fibrosis, cirrhosis, and HCC, were diagnosed based on clinical and radiological parameters (portal hypertension, esophageal/gastric varices, and with ascites as decompensated or otherwise compensated), ultrasonographic evidence, and elevated levels of liver enzymes.

#### 2.1.2 Inclusion criteria

*ALD cases.* A total of 128 consecutive cases belonging to a single ethnicity (North India) with a history of excessive alcohol consumption (characterized as >80 gm/day for >10 years and with attributes of alcohol dependence (25)) were included in this study. Both in-patients or outdoor patients diagnosed with ALD in the Department of Gastroenterology, All India Institute of Medical Sciences, New Delhi, India, were selected as cases. ALD was diagnosed by any of the relevant clinical, radiological and biochemical investigations like, LFT, USG, Upper GI tract Endoscopy. Hepatocellular carcinoma (HCC) was not detected in any subject included in the study. Prior consent from the relative of the patient/patient was undertaken for inclusion in this study.

*Alcoholic and non-alcoholic health controls.* A total of 184 alcoholic control subjects (ALC) and 152 healthy non-alcoholic controls (NALC) of North Indian origin were enrolled in the study, having “significant” alcohol intake or no intake, respectively, but no evidence of liver diseases was detected either in biochemical tests (normal liver function tests) or on ultrasonography, endoscopy, and/or in transient elastography. Individuals with only steatosis or non-alcoholic fatty liver were also included as controls.

#### 2.1.3 Exclusion criteria

Subjects, including cases and controls, were excluded if they had HBsAg; anti-HCV or HIV sero-positivity; presence of diabetes mellitus; evidence for alternative causes for liver diseases such as Wilson’s disease, autoimmune and drug-induced liver diseases; different ethnicity; and failure to give consent. Both ALD or ALC subjects with less than 10 years of alcohol use history were excluded from this study.

### 2.2 Sample collection and genomic DNA isolation

The blood samples were collected with and without anticoagulant (EDTA) from each participant for genomic DNA isolation, biochemical test, and immunoassays. Host genomic DNA was extracted from whole blood using the QIAamp DNA mini kit (Qiagen, Germany) using the Qiasymphony SP automated nucleic acid isolation system. Serum samples separated from blood were used for biochemical tests and immunoassays.

### 2.3 CD 14 gene sequence, primer designing, and SNP genotyping

The CD14 gene was located in the complement strand of chromosome 5q31.3 from positions 140631732 to 140633701 bp (Ref seq: NC_000005.10 and GRCh38.p13 assembly). The gene consists of three exons. The primers were designed for PCR amplification, flanking all three exons and the upstream promoter region (−760 nt upstream, +12 ATG). The primer pairs used for PCR amplification of the CD14 gene promoter (−760 bp upstream), exons 1–3 including introns (**Figure 2A**) were pair 1, −760-5pr-F:AGCAACAGAGCAAGACTCTATC, −421-in1-R: CCAGTACCATACT CTGCACTATC; pair 2: −431-in1-F: CAGAGTATGGTACTGGCCTAAG, +12-ex2-R: CTACACTCA CCATGGTCGATAA; pair 3: −1-ex2-FCATGGTGAGTGTAGGGTCTTG, 482-ex3-R:ATG GTGCCGGTTATCTTTAGG; pair 4, 452-ex3-F: CGCTCGAGGACCTAAAGATAAC, 939-ex3-R: CGACAGATTGAGGGAGTTCAG and pair 5, 844-ex3-F: CTAGACCTCAGCCACAACTC, 1355-ex3-R: GCACATAGCAGACATCCAATAAAG. The PCR-amplified were subjected to both Sanger sequencing and PCR-RFLP using restriction enzyme HaeIII digestion for CD14 polymorphisms (rs2569190). The SNPs reported in the dbSNP database for the promoter region, including exonic and intronic regions were evaluated for North Indian populations.

### 2.4 Biochemical test

Biochemical assays for ALD, ALC and NALC subject(s) were carried out using commercial kits (ERBA Diagnostics, Mannheim, Germany) as per instructions of the manufacturer. Briefly, total bilirubin, total protein, and albumin were estimated by the Diazo End Point, the Biuret End Point, and by the BCG Dye End Point method, respectively. Aspartate transaminase (AST), alanine transaminase (ALT), and alkaline phosphatase (ALP) were estimated by the IFCC (International Federation of Clinical Chemistry) Kinetic method. For AST or ALT activity, 2-Oxoglutarate was used as a substrate which converted to the final product L-Lactate and was measured photometrically at 340 nm. ALP activity was measured for the final product, 4-nitrophenol, at 405 nm.

### 2.5 Immunoassay for sCD14, TLR4, MD2, and cytokines

Serum sCD14, TLR4, MD2, TNFα, IL1β, IL6, IFNγ, IL-10, and IL-4 levels were estimated by enzyme-linked immunosorbent assay (ELISA) with the respective human immunoassay kit (Diaclone SAS, France). The serum LBP was measured using a competitive ELISA kit (R&D Systems, MN, USA). Absorbances were recorded at 450 nm and interpolated using known standard curves.

### 2.6 Droplet digital PCR for serum bacterial DNA concentration

Droplet digital PCR (ddPCR) for serum bacterial DNA concentration was developed in-house. Briefly, a single colony of *E. coli* was grown overnight at 37°C on a shaker and bacterial DNA was isolated. Absolute bacterial DNA copy number (copies/µl) was determined by ddPCR (Biorad) using primer pair for 16S rRNA gene (16S-27F-AAGAGTTTGATCCTGGCTCAG, 16S-244R-CCCACTGCTGCCTCCCGTAG, 357 bp). The bacterial DNA concentration was used as a standard in real-time quantitative PCR (RT-qPCR).The bacterial DNA load in the serum samples of the ALD, ALC, and NALC groups was determined by a RT-qPCR assay interpolating concentration from the standard curve (**Figures 5Ai–v**).

### 2.7 Cloning of CD14 SNP flanking region in promoter-less reporter vector to study its influence on promoter activity

The basal promoter region (−431 to +1 ATG) of the CD14 gene was PCR amplified by primer pair 2 using genomic DNA as a template that was previously confirmed for homozygous (rs2569190, CC or TT) genotype. The basal promoter deletion construct flanking (−760 to −421) was PCR amplified by primer pair 1 using the above mentioned template DNA for both CC and TT genotypes. These four PCR products were cloned into the pGEMT-easy vector. The fragments were further digested with EcoRI and subcloned into the pRL-null vector at the EcoRI site. The direction of clones was checked by Pst I RE digestion and the genotype was further confirmed by Sanger sequencing. The pRL-null forward direction clones of −760 to −421 and −431 to +12 for both CC and TT genotypes were transfected into the Huh-7 hepatoma cell line using Lipofectamine 2000 reagent (Invitrogen). The reporter renilla luciferase activity in transfected cells was measured at 24 h post transfection in a Glomax luminometer using a dual-Luciferase Reporter Assay System (Promega, USA).

### 2.8 Statistical analysis

All the statistical analyses were performed using SPSS (ver. 20.0), R software (ver. 2.0), and GraphPad Prism (ver. 9.0). Associations between alleles/genotypes and disease occurrence were considered “strong” and “statistically significant,” where the p-value was <0.05. For genetic association analysis, allelic and genotypic frequencies were calculated by the direct gene counting method. Initially, the quality of each genotype was tested by Hardy–Weinberg Equilibrium (HWE) testing, which determines whether individual loci or SNPs across the population are in equilibrium or under the influence of any evolutionary forces. Considering the major allele in the control population as reference allele 1 and variant allele 2, the associated risk genotypes were evaluated using dominant (11 + 12 vs. 22), recessive (11 vs. 22) and additive (11 vs. 12 + 22) genetic models. Different biochemical markers or traits like bilirubin, albumin, etc. were also studied to observe whether there was any significant variation in their levels among the patients and controls. The Student’s test, Chi-square test, Kolmogorov–Smirnov test, Kruskal–Wallis test, etc. were done for the analyses wherever needed. To identify the independent predictors of significant liver disease, binary logistic regression analysis using the frequencies of genotypes of rs2569190 of the CD14 gene, amount of total alcohol consumption, age, weight, and height as independent variables was performed.

## 3 Results

### 3.1 Demographic, clinical, and biochemical characteristics of the study population

**Figure 1:**
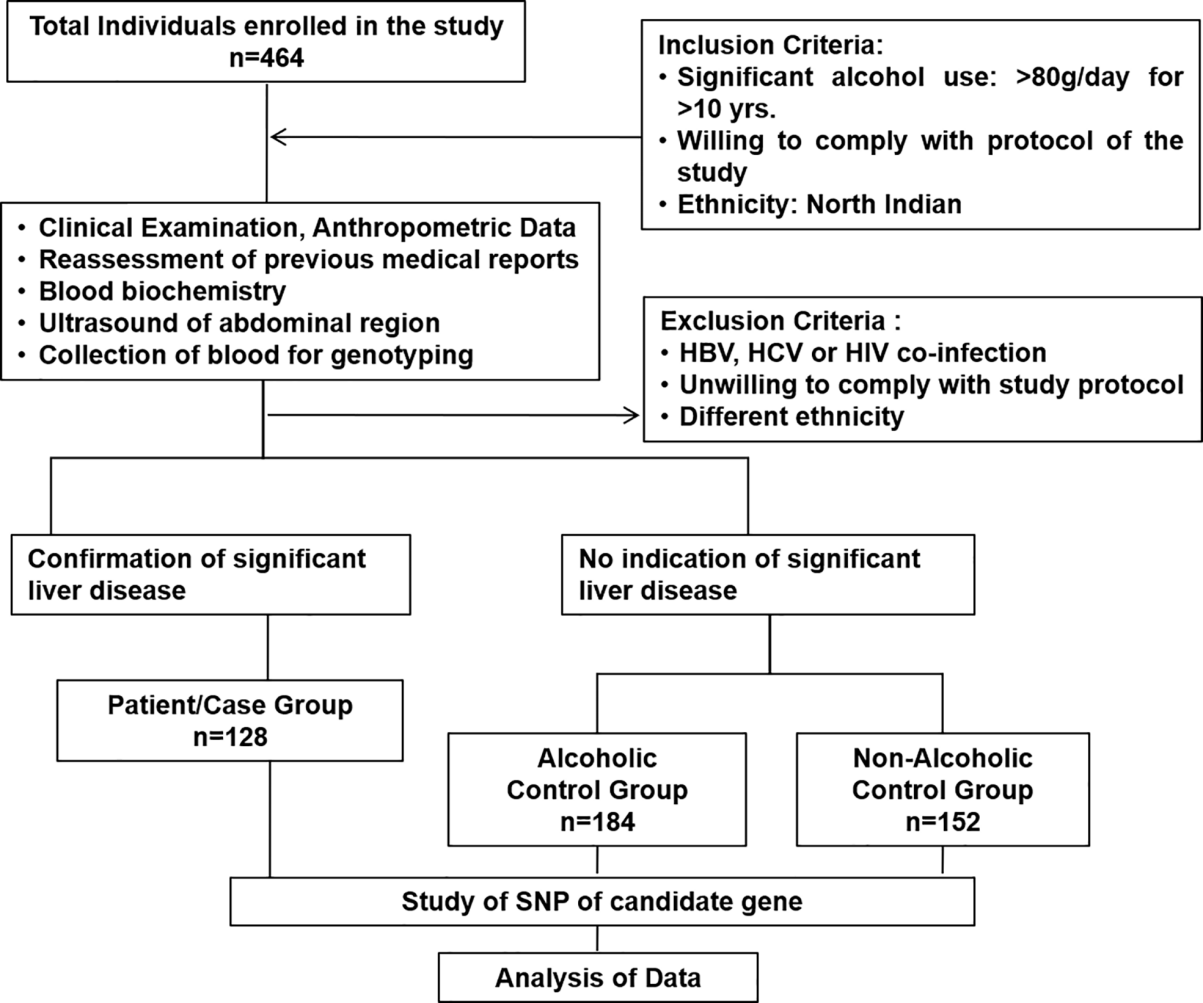
Consort diagram of study cohort with inclusion and exclusion criteria.

The present study included 464 individuals (ALD cases 128, alcoholic control 184, and non-alcoholic control 152) out of 500 screened (**Figure 1**). The study population belonged to a single ethnic group in North India and included primarily men (100% in the patient group and 100% in control groups, respectively). The patients and control groups were comparable in terms of age, gender distribution, and total lifetime alcohol intake. The baseline clinical and biochemical characteristics of the study population are mentioned in **Table 1**. The ALD patients had significantly (P <0.05) higher serum levels of total bilirubin, ALT, AST, ALP, and lower serum levels of total protein, albumin, and hemoglobin as compared to both the ALC and NALC control groups, respectively (**Table 1**). The distributions of these biochemical variables among all three groups were evaluated by the Kolmogorov–Smirnov (KS) test. The KS tests for normality indicated that none of the biochemical markers followed normal distribution among ALD patients while they displayed normal distribution (**Table 1**) among the control groups (except for ALP among the ALC individuals).

**Table 1:** Demographic, clinical and biochemical characteristics among ALD patients, Alcoholic and non-alcoholic Controls.

Sl. No.	Parameters	a. ALD Patients (n = 128)	b. ALC Controls (n = 184)	c. NALC Control (n = 152)	P-value* a vs. b	P-value*a vs. c
**1**	**Gender, Male%**	**100**	**100**	**100**	**–**	**–**
2	Age (years)	45.2 ± 10.0	44.1 ± 8.8	43.9 ± 11	0.305	0.306
3	BMI (kg/m^2^)	20.59 ± 3.04	23.72 ± 3.75	25.48 ± 3.24	**<0.001**	**<0.001**
4	LSM (kPa)	N.A.	7.36 ± 3.53	6.26 ± 2.17	N.A.	N.A.
5	Total Bilirubin (mg/dl)	4.41 ± 5.33	1.11 ± 0.26	0.57 ± 0.03	***<0.0001***	***<0.0001***
6	Total Protein (g/dl)	6.83 ± 0.86	7.46 ± 0.52	7.32 ± 0.65	***<0.0001***	***<0.0001***
7	Albumin (g/dl)	3.28 ± 0.95	4.34 ± 0.38	4.36 ± 0.45	***<0.0001***	***<0.0001***
8	AST (IU/L)	99.12 ± 119.34	24.95 ± 5.29	25.95 ± 5.55	***<0.0001***	***<0.0001***
9	ALT (IU/L)	60.45 ± 83.43	32.70 ± 7.83	32.59 ± 10.33	***<0.0001***	***<0.0001***
10	ALP (IU/L)	300.86 ± 203.63	124.06 ± 22.42	141.03 ± 64.94	***<0.0001***	***<0.0001***
11	Haemoglobin (g/dl)	10.87 ± 2.69	14.1 ± 0.76	13.8 ± 1.24	***<0.0001***	***<0.0001***
**Kolmogorov–Smirnov test to compare distribution of biochemical variables among the ALD patients, ALC, and NALC controls**						
		Kolmogorov–Smirnov Z, P-value**	Kolmogorov–Smirnov Z, P-value	Kolmogorov–Smirnov Z, P-value		
	Total Bilirubin (mg/dl)	**3.53, 0.0001**	1.17, 0.124	0.93, 0.316		
	Albumin (g/dl)	**1.45, 0.030**	0.51, 0.959	1.04, 0.226		
	AST (IU/L)	**2.96, 0.0001**	0.71, 0.703	1.08, 0.194		
	ALT (IU/L)	**3.55, 0.0001**	1.04, 0.231	0.92, 0.368		
	ALKP (IU/L)	**2.56, 0.0001**	**3.19, 0.0001**	0.62, 0.972		

**p-value obtained from Student’s t-test; Values are Mean ± SD.

**p-values obtained from Kolmogorov–Smirnov test for normality.

*p-value obtained from Student’s t-test; Values are Mean ± SD.

BMI, Body mass Index; LSM, Liver stiffness measurement; AST, Aspartate Transaminase; ALT, Alanine Transaminase; ALP, Alkaline Phosphatase.

Bold values are statistically significant.

NA, Not available.

### 3.2 CD14 gene structure and single nucleotide polymorphisms

**Figure 2:**
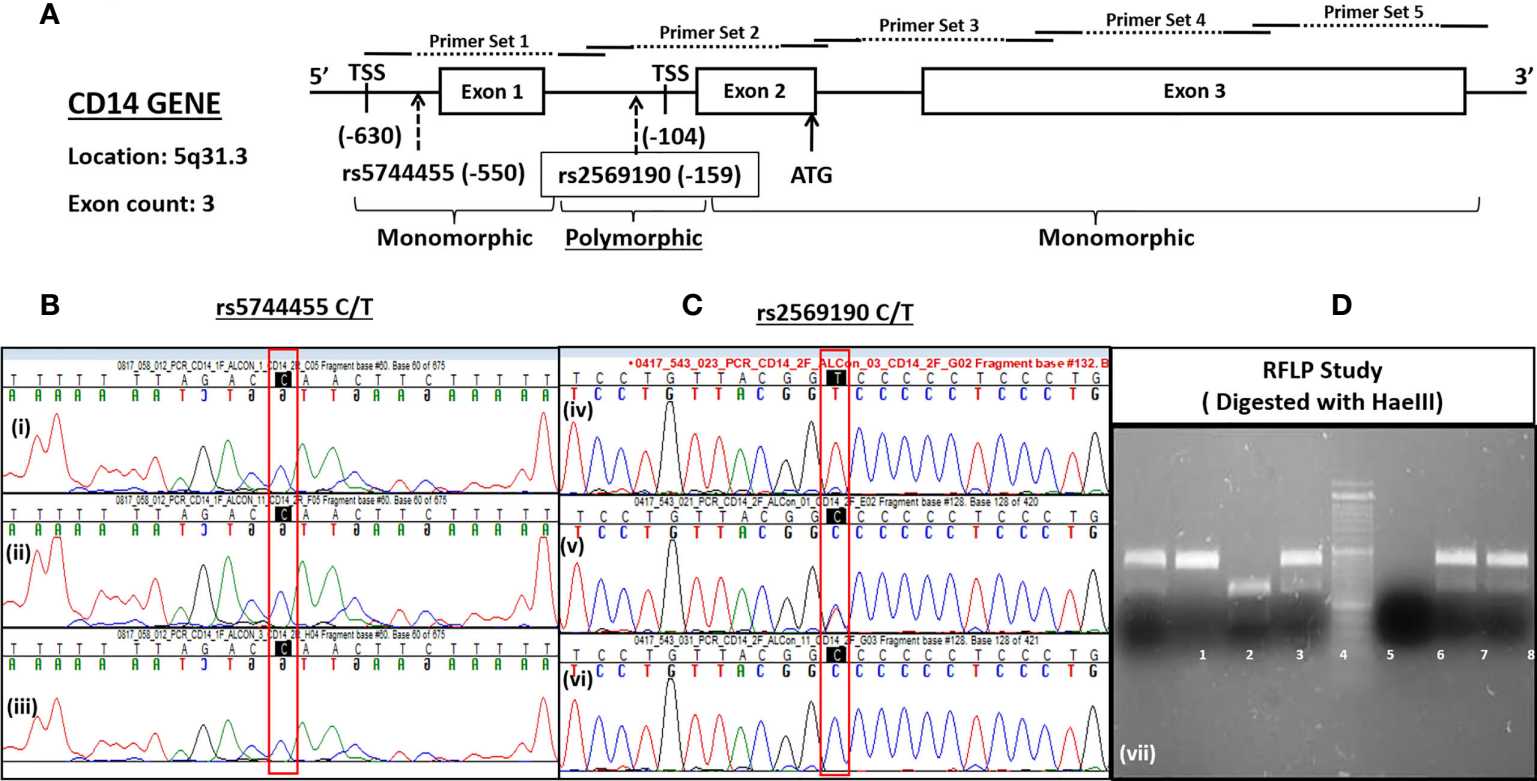
Schematics of CD14 gene **(A)** and its SNP genotyping by sequencing **(B, C)** and RFLP **(D)**.

The CD14 gene consists of three exons that encode four transcript variants (**Figure 2A**). Transcription initiation of predominant transcript variant 1 (Ref: NM_000591, 1,356 nt) starts at −104 bp upstream (TSS-104) of the start codon (ATG), which lies at the end of exon 2. Transcription initiation of the other three transcript variant (NM_001040021, 1,544 nt; NM_001174104, 1,644 nt; and NM_001174105, 1,523 nt) begins at −630 bp upstream (TSS-630) of the start codon or at the beginning of exon 1. Therefore, the intronic region between exons 1 and 2 may serve as a promoter, including the upstream genome region of exon 1. We have evaluated this intronic region (−431 nt to +1 ATG) for promoter activity by cloning it in a promoter-less renilla luciferase reporter vector and studied the influence of polymorphisms on CD14 gene expression.

There are >1,000 SNPs reported for the CD14 gene (exonic/intronic and flanking regions), but only a few of them show ~50% heterozygosity as mentioned in the NCBI dbSNP database (https://www.ncbi.nlm.nih.gov/SNP/snp_ref.cgi?locusId=929). The minor allele frequency (>0.2) submitted with 1,000 genome data for polymorphisms at promoter regions are rs2569192, 0.2149; rs3138076, 0.2440; rs2915863, 0.3976; rs3138078, 0.2430; rs2569191, 0.4748; rs577487266, 0.0002; rs572215387, 0.4511; rs112865855, 0.0417; rs5744455, 0.2015, and rs2569190, 0.4688. The genetic association of these SNPs in the promoter region was evaluated for north Indian populations.

### 3.3 Frequency distribution of CD14 SNP genotypes/alleles and Hardy–Weinberg equilibrium testing

The CD14 upstream promoter region (−760 to +12 ATG) was initially PCR amplified and sequenced for genotyping of SNPs such as rs572215387, rs5744455, and rs2569190. Most SNPs, including rs5744455, were monomorphic (**Figure 2B**), whereas the SNP rs2569190 was polymorphic (**Figure 2C**). The rs2569190 SNP was genotyped by PCR-RFLP using Hae III restriction enzyme and the accuracy of the variants detected by RFLP was confirmed by sequencing of randomly selected 5% of total samples (**Figures 2C, D**). The frequency of minor allele (T) and observed genotype percentages for SNP rs2569190 in both case (ALD) and control (ALC and NALC) populations are mentioned in **Table 2A**. An increased frequency of risk genotype (TT, 29.7%) and risk allele (T, 0.547) was observed for ALD cases as compared to controls (TT genotype: NALC, 25.6%, ALC, 22.8%; T allele: NALC, 0.454, ALC, 0.443). Expected genotypes were derived from observed genotype frequencies and the test of deviations from Hardy–Weinberg equilibrium (HWE) in both case (ALD) and control (ALC and NALC) populations were evaluated by a chi-square test using observed and expected genotype frequencies. The test of deviation from HWE was not found to be significant for ALD cases (p = 0.92) and ALC controls (0.07), but it was significant for NALC control (P = 0.01), as mentioned in **Table 2A**. The SNP loci were not influenced by evolutionary forces (mutation, genetic drift, and migration) if HWE was not found significant and suitable for genetic association studies.

**Table 2:** Frequency distribution for CD14 gene SNP (rs2569190) genotype/minor allele and its genetic association for Alcoholic liver disease.

Gene/SNP	Genotype, Minor allele	Case (a)ALD, %n = 128	HWEχ^2^P-value		Control (b)ALC, %n = 184	HWEP-value	Control (c)NALC, %n = 152	HWEP-value
CD14 rs2569190 (C/T)	CC CT TT T	26 (20.3) 64 (50.0) 38 (29.7) 0.547	χ^2^, 0.010 p, 0.920		63 (34.3) 79 (42.9) 42 (22.8) 0.443	χ^2^, 3.108 p, 0.078	53 (34.9) 60 (39.5) 39 (25.6) 0.454	χ^2^, 6.311 p, 0.012
**a. Test for deviations from Hardy–Weinberg equilibrium**								
**Gene/SNP**	**Genotype,Minor allele**	**ALD vs. ALCOR (95% CI)**	**P-value**	**ALD vs. NALCOR (95% CI)**		**P-value**	**ALD vs. ALC + NALCOR (95% CI)**	**P-value**
CD14 rs2569190 (C/T)	CC CT TT CT+TT C T	Ref 1.96 [1.12–3.45] 2.19 [1.16–4.13] 2.04 [1.21–3.46] Ref 1.52 [1.10–2.09]	0.018 0.014 0.007 0.011	Ref 2.17 [1.21–3.91] 1.99 [1.04–3.80] 2.10 [1.22–3.62] Ref 1.45 [1.04–2.03]		0.009 0.037 0.007 0.028	Ref 2.05 [1.22–3.45] 2.09 [1.18–3.72] 2.07 [1.27–3.36] Ref 1.48 [1.11–1.98]	0.006 0.011 0.003 0.007
**b. Test for genetic association**								

*OR, 95% CI and p-value obtained from 2 × 2 Chi-square test for Association.

Bold values are statistically significant.

### 3.4 Genetic association of CD14 SNP (rs2569190) with alcoholic liver disease

The genetic association of the SNP rs2569190 with the risk of developing ALD was done by evaluating the odds ratio (OR) of genotypes and alleles between ALD cases and ALC and NALC control populations. The dominant allele C and genotype CC of the SNP rs2569190 were considered as reference and a subsequent association study was done by calculating the OR for risk allele T and genotype (TT, CT, and CT + TT) as mentioned in **Table 2B**. Significant genetic associations of risk genotype (TT) and allele (T) were observed with OR >1 for ALD cases when compared with ALC, NALC, and ALC + NALC controls (**Table 2B**).

### 3.5 Effect of associated SNP rs2569190 risk genotype (TT) on biochemical traits/variables

The impact of CD14 SNP risk genotypes on pathological levels of biochemical variables was evaluated by performing Kruskal–Wallis (KW) analysis. These variables did not follow the normal distribution for ALD patients (**Table 1**). A Kruskal–Wallis (KW) analysis of variance test across the rs2569190 genotypes was carried out based on both the unadjusted biochemical variables as well as the variables that were adjusted for covariates like age, BMI, and total alcohol exposure. Evidently, the negation of such a test would pinion an association of the risk genotype with the biochemical variable. From the KW test, the effect of rs2569190 SNP genotypes (CC/CT/TT) on increased level total bilirubin, albumin, and AST level was found significant (p = 0.048, 0.005, and 0.000) with the unadjusted data and also remained significant (p = 0.049, 0.006, and 0.000) even when the adjusted data were used. Similarly, the degree of significance for association with the reduced level of albumin and enhanced level of AST was maintained after performing the KW test for both the unadjusted and adjusted datasets. No significance was observed for ALT and ALP in both the unadjusted (p = 0.420 and 0.272) and adjusted (p = 0.437 and 0.280) datasets.

### 3.6 Univariate and multivariate analysis for identifying independent predictors of liver disease in AUD

Group-wise analysis with binary logistic regression in this cohort (n = 484), detected total bilirubin and albumin as predictors for the development of significant liver disease (p-value = 0.006 and 0.042, respectively) when compared between the ALD and ALC groups (**Table 3**). Similarly, when ALD and NALC groups were compared, total protein (p-value = 0.032), alkaline phosphatase (p-value = 0.001) and hemoglobin (p-value = 0.014) were found to be serving as independent predictors for the severity of liver disease (**Table 3**).

**Table 3:** Multivariate analysis in ALD patients and control groups for the severity of liver disease.

Biochemical variables	Odd’s Ratio (OR)	95% CI	p-value	Correlation with severity of disease
**ALD patients vs. Alcoholic Control Group**				
Total Protein	0.23	0.06–0.88	0.032	**↓**
Alkaline Phosphatase	1.05	1.03–1.06	0.001	**↑**
Haemoglobin	0.55	0.35–0.89	0.014	**↓**
**ALD patients vs. Non-Alcoholic Control Group**				
Total Bilirubin	909.93	112.86–7,336.33	0.001	**↑**
Albumin	0.061	0.02–0.16	0.001	**↓**

↑, increase; ↓, decrease.

### 3.7 Comparison of soluble CD14, LBP, MD2, TLR4, and cytokine levels in the serum among AUD patients and healthy control

**Figure 3:**
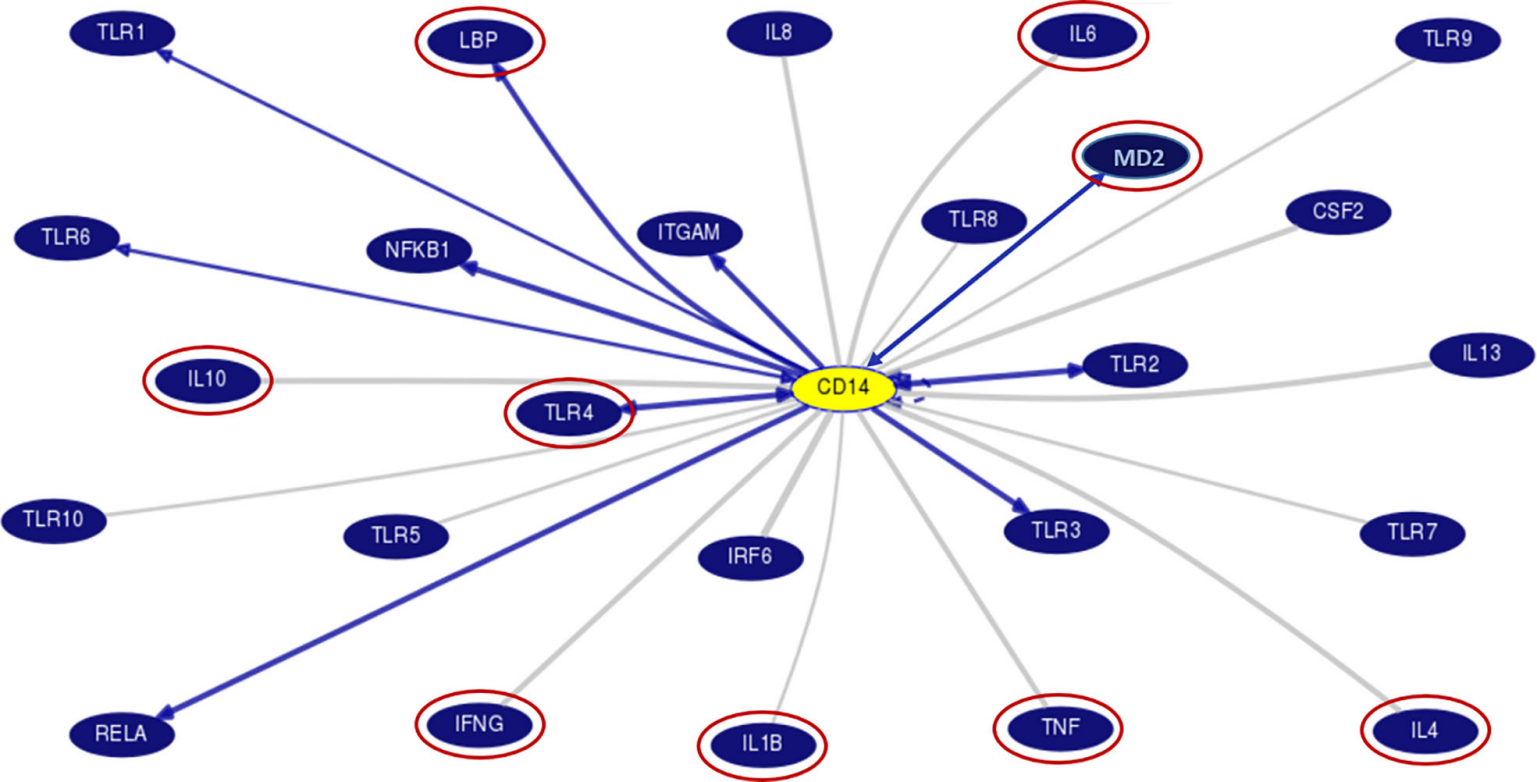
Gene interaction model of CD14 gene with other genes as generated/adapted by using “Gene Interactions” track of UCSC genome browser (www.genome.ucsc.edu) based on curated pathway/protein-interaction databases and interactions found through text mining of PubMed articles (https://genome.ucsc.edu/goldenPath/help/hgGeneGraph.html). The interaction of CD14 with other proteins is arrow marked. The red circle proteins are studied in the serum of patient samples. CD14, Cluster of differentiation 14, CSF2, Colony stimulating factor 2; IFN, Interferon; IL, Interleukins; IRF6, Interferon Regulatory Factor 6; ITGAM, Integrin Subunit Alpha M; LBP, Lipopolysaccharide binding protein; MD2, Myeloid Differentiation factor 2; NFKB1, Nuclear Factor Kappa B Subunit 1; RELA, REL-associated protein; TLR, Toll-like receptors; TNF, Tumor Necrosis Factor.

Chronic alcohol consumption resulting in leaky gut syndrome and translocation of bacterial endotoxin (LPS) is attributed to chronic inflammation and severe liver diseases. Recognition of LPS by LBP, CD14, and the TLR4/MD2 complex activates an inflammatory cytokine cascade. The CD14 protein interaction with other molecules was curated using UCSC genome browser tools for gene interaction as depicted in **Figure 3**. We have determined serum levels of these molecules and cytokines among alcoholics with or without liver disease and healthy non-alcoholic control groups (**Table 4**). Significantly higher levels of sCD14, LBP, TLR4, and inflammatory cytokines were observed in ALD patients when compared with NALC control (**Table 4**). Anti-inflammatory cytokine IL-4 and MD2 levels were significantly lower in ALD groups than NALC group. IL-10 levels were not found to be significant among groups. Comparisons of serum levels in ALD patients vs. ALC control showed significant levels for CD14, TNFα, IL1β, and IL6 (**Table 4**).

**Table 4:** Expression of CD14 and other pro- and anti-inflammatory cytokines in sera of individuals of the ALD, ALC, and NALC groups.

Sl. No,	Parameters	a. ALD Patients(n = 128)	b. Alcoholic Controls (n = 184)	c. Non-Alcoholic Control (n = 152)	P-value* a vs. b	P-value*a vs. c
1	CD14 (ng/ml)	267.98 ± 223.65	197.72 ± 115.11	152.11 ± 43.99	**0.033**	**<0.0001**
2	LBP (ng/ml)	156.12 ± 128.47	131.68 ± 69.58	107.23 ± 45.91	0.198	**0.006**
3	MD2 (ng/ml)	1,212.35 ± 735.67	1,513.09 ± 1,108.69	2,055.79 ± 1,365.81	0.083	**<0.0001**
4	TLR-4 (ng/ml)	4.01 ± 1.49	3.55 ± 2.07	2.89 ± 1.70	0.165	**<0.0001**
5	TNFα (pg/ml)	199.70 ± 40.04	97.01 ± 15.16	20.21 ± 4.99	**<0.0001**	**<0.0001**
6	IFNγ (pg/ml)	4.81 ± 3.81	4.40 ± 1.93	3.20 ± 0.98	0.459	**0.002**
7	IL-1β (pg/ml)	237.03 ± 70.23	98.78 ± 12.64	57.67 ± 6.16	**<0.0001**	**<0.0001**
8	IL-4 (pg/ml)	0.95 ± 0.14	0.98 ± 0.12	1.17 ± 0.18	0.210	**<0.001**
9	IL-6 (pg/ml)	3.89 ± 2.06	1.22 ± 0.83	0.71 ± 0.39	**<0.0001**	**<0.0001**
10	IL-10 (pg/ml)	8.77 ± 4.73	7.35 ± 3.93	9.82 ± 3.38	0.076	0.164

*p-value obtained from Student’s t-test. Bold values are statistically significant.

### 3.8 The influence of the CD14 (−159 C/T) SNP on sCD14 and downstream cytokine levels in the serum of ALD patients and control

**Figure 4:**
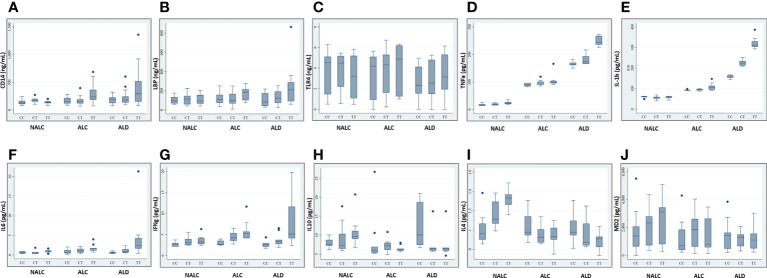
Expression of CD14 **(A)**, LBP **(B)**, TLR4 **(C)**, TNFa **(D)**, IL-1b **(E)**, IL6 **(F)**, IFNg **(G)**, IL10 **(H)**, IL4 **(I)**, and MD2 **(J)** in sera of individuals of genotype CC/CT/TT of rs2569190 among the ALD, ALC, and NALC groups. CD14, Cluster of differentiation 14; LBP, Lipopolysaccharide binding protein; TLR, Toll-like receptors; TNF, Tumor Necrosis Factor; IL, Interleukins; IFN, Interferon; MD2, Myeloid Differentiation factor 2; NALC, Non-alcoholic healthy controls; ALC, Alcoholic control; ALD, Alcoholic liver disease. Outliers are mentioned as (●).

Recent studies investigating sCD14 levels in children recruited from the general population or ulcerative colitis patients found an association between sCD14 serum levels and CD14 SNP genotypes (23, 26). Any such association with ALD patients vs. controls was investigated by determining these levels in an equal number of individuals for respective genotypes (CC/CT/TT) of ALD, ALC, and NALC groups. Regardless of whether they belonged to the ALD patient or control groups, CC genotypes and, to a lesser extent, CT genotypes, had lower levels of sCD14 than TT carriers (**Figure 4A**). A similar scenario was observed for the other pro-inflammatory cytokines (LBP, TLR4, TNFα, IL1β, IL6, and IFNγ) in TT genotype carriers those exhibited increased serum levels than the CC and CT individuals of the ALD patient group as well as the control groups (**Figures 4B–G**). Whereas, anti-inflammatory cytokines (IL10, IL4) and MD2 levels in the individuals of CC genotype in the ALD patients and ALC group showed higher expression than CT or TT genotype carriers of each group respectively (**Figures 4H–J**).

### 3.9 Serum bacterial DNA load among the CC, CT, and TT genotypes of CD14 rs2569190 AUD patients and healthy control

**Figure 5:**
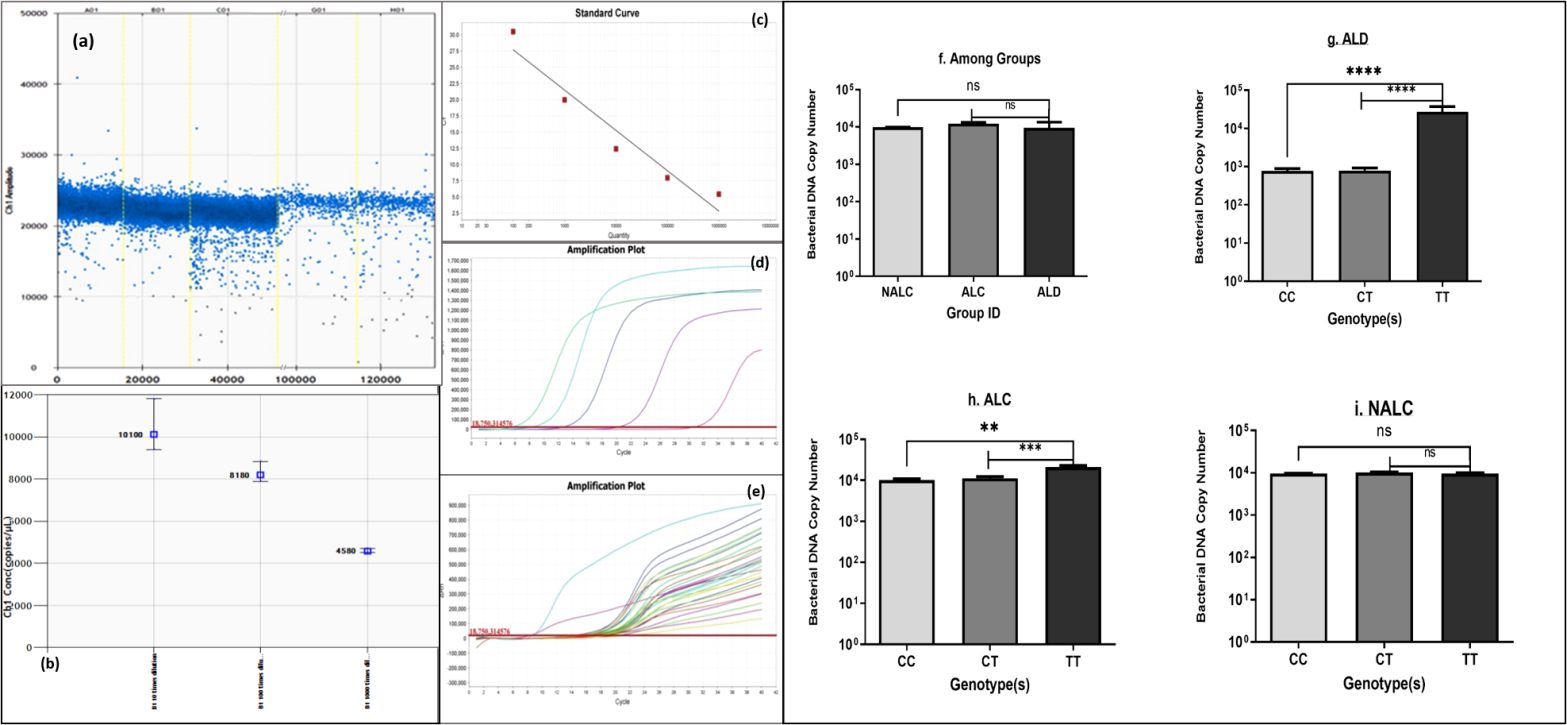
Bacterial DNA copy number **(A, B)** generated by ddPCR, **(C–E)** of each individual calculated from standard curve generated by real-time PCR, **(F)** of the three groups and **(G–I)** of genotypes CC/CT/TT of ALD, ALC, and NALC groups respectively. Significant differences p >0.05 was observed for ALD and ALC group represented with * in the scale. **p value =0.001, ***p value =0.0001, ****p value =0.00001, ns, not significant.

Comparison of bacterial DNA concentrations in ALD, ALC, and NALC groups or across different genotypes of these groups was carried out by quantitative real-time PCR (**Figures 5C–E**). Bacterial DNA copy numbers were significantly higher in TT genotype carrier groups of ALD patients (p = 0.0008) and ALC groups (p = 0.003) than the corresponding CC and CT carriers (**Figures 5G, H**). On the other hand, no significant (p = 0.3301) difference was observed among the three genotype (CC/CT/TT) carriers of the NALC group (**Figure 5I**).

### 3.10. Influence of polymorphisms on CD14 promoter activity

**Figure 6:**
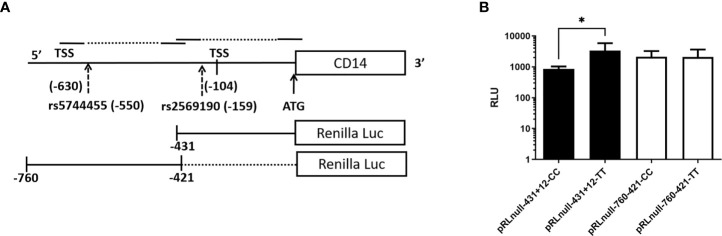
Renilla luciferase reporter constructs and the effect of polymorphisms affecting promoter activity. **(A)** Schematics of the promoter region and SNP positions **(B)** Effect of polymorphisms on promoter activity. TSS, Transcription start site; Luc, Luciferase; RLU, Relative Luminescence Unit, *Significant p-value, 0.03.

The SNP rs2569190 (C/T) lies in the intronic region of CD 14 exon 1 and exon 2, which is −159 nt upstream of the start codon CD14 ORF (**Figure 2A**). The transcription start sites (TSS) of transcript variant 1 are predicted to be −104 nt in the intron, whereas the TSS for transcript variants 2–4 is predicted to be −630 nt. The region −431 to +12 ATG was subcloned in the pRL-null reporter vector to study the effect of the rs2569190 SNP on promoter activity, and the region −760 to −421 nt served as control (**Figure 6A**). The promoter activity was observed for the intronic regions −431 to +12 ATG and −760 to −421 region (**Figure 6B**). But the influence of SNP rs2569190 (C/T) was observed for the −431 to +12 region where promoter activity for the TT genotype (3,338 ± 2,466) was significantly (p = 0.03) higher than the CC genotype (851.3 ± 430.2). No significant difference in promoter activity was observed when these SNP regions were deleted in the construct pRL-null −760 to −421 (**Figure 6B**).

## 4 Discussion

The fast progress of alcohol per capita consumption in the Asian subcontinent over the past decade has led to a rising emphasis on regional scientific attempts to understand the problem (WHO, 2002). From the Indian cultural point of view, the perspective on alcohol use in India has always been highly ambivalent (27). The common belief of alcohol use being a male-predominated health hazard in the Indian subcontinent (28, 29), is exhibited by the predominate male distribution in our present study. There is limited data of help seeking among females which may be due to the cultural and social outlooks (like, excessive stigma about the use of alcohol by females) that may ultimately deter them from reporting to the hospital (30). The present study was designed to explore an association between genetic polymorphisms and the disease individually on a backdrop of alcohol use disorders, thus ALD patients, patients with alcohol use disorders without liver disease, and individuals without alcohol use (NALC) were enrolled. Proper clinical investigations, like biochemical assays, ultrasonography, endoscopy, and fibroscan (LSM <11.3) were used rightfully to define “alcoholics with ALD” and the control groups (ALC and NALC) (31, 32).

Alcoholic liver disease is a complex disease that results due to a culmination of the toxic effects of alcohol, the environment, and the host genetic milieu. Alcohol stimulates a pro-inflammatory condition in the liver where the outcomes of inflammatory cytokine genes play a pathogenic role (33). The polymorphisms in these genes and their influences are not well studied in the outline of Indian ethnicity. On the basis of this circumstance, our aim was to investigate the influence of CD14 gene polymorphisms on the risk of development of significant liver disease due to excess alcohol exposure in the North Indian ethnic population. One of the most studied genes in the inflammatory pathway is the CD14 gene and its polymorphisms (23). In our study, an increased frequency of risk genotype (TT, 29.7%) and risk allele (T, 0.54) was observed for ALD cases as compared to controls (TT genotype: NALC, 25.6%, ALC, 22.8%; T allele: NALC, 0.45, ALC, 0.44) with significant genetic association (**Table 2**). The T allele was reported as a minor allele in the 1,000 genome database with a frequency of 0.46, which corresponds to our control (ALC and NALC) population. Mancina et al. have shown association of this CD14 rs2569190 SNP with alcoholic cirrhosis in male from Rome, Italy and T allele frequency was higher in alcoholics (0.58) than non-alcoholic control (0.48) (34). Another prior study from Finland was coherent with our study assenting that advanced ALD was lot more frequent among patients with T allele SNP rs2569190 (22). However, the meta-analysis of eight studies from Greece, Czech, Germany, Portugal, China, UK, and Finland did not find significant association of this SNP when ALD patients were compared with healthy. However, no significant association was observed in the heterozygous comparison (TC vs. CC) and dominant genetic model (TT/CT vs. CC) for ALD cases vs. alcoholics without ALD control. But, there was no significantly greater of T allele frequency for ALD cases (0.48) than controls (NALC, 0.46 and ALC, 0.45) due to the mixed ethnic population (35). A previous study from the North Indian population did not find any association with alcoholics and the T allele frequency was observed to be more or equivalent in both healthy control (0.64) and alcoholics (0.67) (36). It is clearly evident that association of TT genotype and T allele can occur in some ethnic populations depending upon its frequency in the population, whereas the pooled genomic data of multiple ethnic populations may favor biased results requiring focused ethnic population-based study. In India, several case–control association studies were conducted representing different regions/states for CD14 SNP rs2569190 (C/T) in different inflammatory diseases by comparing them with the healthy control population. The frequency of minor T alleles in healthy controls has shown differences across the region, indicating multi-ethnic variations within India. The minor T allele frequencies in this study population and their significant association with different diseases are mentioned. The recent study from the eastern part (Odisha state) of India in all female subjects has shown an association of rs2569190 (C/T) SNP to increased predisposition to lupus (SLE) and the T-allele frequency was 0.46 for healthy control and 0.58 for SLE cases (37). The MAF (T allele) in other Indian studies are: West Bengal (38), 0.53, control vs. 0.49, Asthma case; Chennai (39), 0.43 in Control vs. 0.54 in Chronic periodontitis case; Hyderabad, Andhra Pradesh (40), 0.41 in Control vs. 0.53 in Ulcerative Colitis case; Chandigarh (41), 0.5 in Control vs. 0.64 in NAFLD case; and Lucknow, UP (42), 0.65 in healthy Control vs. 0.54 in CAD cases. This is a clear indication of an increasing trend of minor T allele frequency from south-eastern to north-Indian population. The T allele is also considered as a major allele whereas the C allele is a risk for some diseases (e.g., CAD) in the North Indian population, but the majority of the healthy population have T as a minor allele for SNP rs2569190 (C/T). For significant genetic association of CD14 SNP in different ethnic groups, an important role is being played by sociological or/with geological differences for allele frequency variations.

The exact modus operandi by which the CD14 −159 (C/T) polymorphism contributes to ALD pathogenesis is not fully comprehended yet. The CD14 −159T allele is transcriptionally more active than the CD14 −159C allele (43). However, this SNP lies in the intronic or putative promoter region. The endotoxin-CD14-mediated activation of Kupffer cells may stimulates cascade of pro-inflammatory mediators excess production including TNFα, IL1β, IL6, IFNγ, and reduced anti-inflammatory cytokines expression including IL10, IL4 for developing ALD (44). The most straightforward and precise way to validate this fact is by assessing the levels of the cytokines in circulation. In our study, soluble CD14 levels as well as LBP, TLR4, TNFα, IL1β, IL6, and IFNγ levels were found to be higher among the ALD patients than the ALC and NALC controls. TNFα and IL1β are crucial mediators of the inflammatory response. Increased CD14 expression (45) with enhanced TNFα and IL1β synthesis plays a vital role in mediating cellular activation and inflammatory processes (46) (**Figure 3**). Elevated levels of TNFα and IL1β were found in our study, and these elevated levels are correlated with poor prognosis in ALD patients (47). Likewise, elevated levels of immunoproliferative cytokines like IL1β, as noted in our study (**Figure 4E**), have been reported to have a crucial role in immune response among ALD patients (48). A similar finding was reported in a Spanish cohort where IL1β was more represented in alcoholic patients than in the control group (49). IL6, another inflammatory cytokine product, is also induced upon stimulation of TLR-4 by LPS or upon stimulation of cells by IL1β or TNFα (50). In our study, the IL6 level was found to be increased quite significantly in the ALD patients (**Figure 4F**), particularly in the carriers of the CD14 rs2569190 TT genotype (**Figure 4F**). In the liver, IL6 is an important stimulator of acute phase response and defense against infection. Persistent activation of the IL6 signaling pathway is injurious to the liver and might even lead to the development of liver cancer (51). Similarly, IFNγ, a critical and pleiotropic cytokine, seems to contribute extensively to the inflammatory response. It is critical for efficient innate and adaptive immune responses and plays a detrimental role in the initiation and/or maintenance of pro-inflammatory activation (52, 53). However, the exact role of IFNγ in the development of liver injury is not yet clearly understood. Previous studies mainly concentrated on assessing the contribution of IFNγ to acute liver and intestinal injuries in animal models, revealing that IFNγ was crucial in aggravating liver injury induced by lipopolysaccharide (LPS) (54) and that it had a key role in causing intestinal inflammation induced by interleukins (55). Accordingly, in our present study, IFNγ was found to be expressed most strongly among the ALD patients with TT genotype (**Figure 4G**) and also to some extent among the TT genotype carriers of alcoholic control individuals (**Figure 4G**). This increased level of IFNγ might be due to the elevated magnitude of endotoxins or LPS present in the portal circulation of those subjects, also evident from the higher levels of LBP (**Figure 4B**) in them.

Conversely, production of IL10, an anti-inflammatory and hepatoprotective cytokine secreted by monocytes and macrophages is reduced in patients with ALD, which is apparent from our present study. Expression of IL10 was comparatively very low among the ALD individuals with the risk genotype (rs2569190-TT) (**Figure 4H**) and was found to be highest among the CC carriers of ALD patients and TT carriers of the non-alcoholic control (NALC) group (**Figure 4H**). IL10 is produced in the liver by the activation of the innate immune system and it inhibits alcoholic liver inflammation *via* activation of its downstream signaling molecule, Signal Transducer and Activator of Transcription 3 (STAT3) in Kupffer cells (56). It can potentially counterbalance the detrimental effects of excessive pro-inflammatory cytokine production, which is substantially reduced in ALD patients. A similar type of observation was also made for the expression level of interleukin-4 (IL4) in our study (**Figure 4J**). Maximum expression of IL4 was observed among the ALD patients with the rs2569190CC genotype and among the TT carriers of the NALC group. IL4 is a multifunctional anti-inflammatory cytokine and is one of the most frequently studied cytokines in inflammation-mediated diseases (57). This intermediary molecule is mainly secreted by activated T helper 2 (Th2) cells and has been reported to influence an individual’s susceptibility to liver disease (58). In many circumstances, IL4 affects the macrophages in a similar way to that of IL10 and prevents the production of pro-inflammatory molecules that are usually induced by IFNγ alone or in synergy with other stimulants of inflammation like TNFα, IL1β, etc. (59).

In this study, we have also looked into another accessory glycoprotein with a pertinent role in LPS responses, namely, Myeloid Differentiation factor 2 (MD2) or Lymphocyte antigen 96 (LY96). It binds to the extracellular domain of TLR4 to develop a functional signaling receptor for bacterial LPS (60, 61). Generally, the binding of LPS initiates the formation of a receptor multimer composed of two copies of the TLR4–MD2–LPS complex (62) which subsequently activates a downstream signaling deluge, resulting in the initiation of transcription factors such as NF-kB and the IFN regulatory factors, ultimately inducing various immune and inflammatory genes. Therefore, along with the enhanced production of CD14, LBP, and TLR4 (**Figures 4A–C**), an increased level of MD2 should be noted in the blood plasma. But, interestingly like IL10 and IL4, MD2 level was found to be significantly low in the blood plasma of the -159TT carriers of ALD group as compared to that of NALC group respectively (**Figure 4K**). MD2 exists in two forms: the membrane-bound form that is associated with the formation of the TLR4–MD2–LPS complex on the cell surface of resident macrophages and the soluble form that is found in plasma (63) and is easily quantified. In 2010, Gray and his group identified an alternatively spliced isoform of human MD2, and termed it MD2 short (MD2s) that is mostly present in the plasma. It lacks the region encoded by exon 2 of the MD2 gene; however like normal MD2, it is glycosylated and secreted and interacts with LPS and TLR4 but is incapable of mediating NF-kB activation and IL-8 production after LPS exposure (63). Therefore, MD2, present mainly in plasma, can be considered as an important negative regulatory component and negative feedback inhibitor of the LPS-mediated TLR4 signaling pathway.

Alcohol-induced liver injury is one of the most common causes of cirrhosis. Therefore, we wanted to find whether different biochemical variables could act as predictors for the severity of liver disease among the ALD patients in comparison to the control groups (**Table 3**). Levels of total protein and alkaline phosphatase were found to increase, while hemoglobin was decreased with the severity of the disease. Similarly, total bilirubin and albumin were also identified as individual predictors of the severity of liver ailment in ALD as compared to the control group (**Table 3**). We have also delineated whether the variants of -159C/T (rs2569190) of the CD14 gene are associated with elevated levels of biochemical indices of alcoholic liver damage or not. Along with genetic factors, other damaging factors like alcohol exposure over a long period of time, BMI, and age affect the severity of liver damage in alcoholic liver disease (64). The rs2569190 SNP genotypes (CC/CT/TT) have shown significant association with enhanced expression of total bilirubin and AST and a reduced level of albumin when the influence of age, BMI, and total alcohol consumption were excluded, which definitely implies that the genotypes of the CD14 gene might actually modulate the level of the afore-mentioned biochemical indices in the study population. Since CD14 has a central role in the innate immune system, the regulation of its expression has been well studied.

In a transient transfection assay, a region of 227-bp upstream of the main transcription start site of CD14 was identified to drive maximal reporter activity (65). Binding sites for Sp1 and members of the CCAAT/enhancer-binding protein (C/EBP) family of transcription factors are present in this region. The integrity of Sp1 and C/EBP motifs located at positions -110 and -135 of the CD14 gene majorly affects the basal promoter activity of the gene. The same C/EBP site was also involved in the TGF-β-dependent activation of CD14 expression (66). Enhanced TGF-β was found to be significantly associated with the risk of ALD in the Bengali population (25). The basal promoter region flanking SNP-159C/T (rs2569190) has shown promoter activity. The region with the TT genotype has shown higher reporter activity than the CC genotype (**Figure 6B**). Besides inflammatory responses, increased expression TGF-β and reduced albumin synthesis were found to be synchronized in hepatic regeneration and hepatic fibrosis in a murine model (67). TGF-β triggers the Smad2‐ and Smad3‐dependent expression of negative regulators of receptor-mediated endocytosis, which subsequently leads to the prevention of reabsorption of important proteins like albumin (68).

Chronic exposure to alcohol results in a highly permeable gut, which gives easy access to microorganisms and endotoxins to travel straight-forwardly in the blood. Besides this, whether bacterial DNA gets access to the circulation due to gut permeability was also tested. Detection of bacterial DNA number in circulation was detected droplet-digital PCR (ddPCR) and quantitative real-time PCR methods (**Figures 5A–E**). Overall, bacterial DNA copy number among the three groups (**Figure 5F**) did not provide a clear view of the differences. When serum bacterial DNA load was determined across genotype (CC/CT/TT), ALD patients showed more bacterial DNA in circulation for the TT genotype.

In conclusion, we are the first to show an association of CD14 polymorphisms with ALD for the Indian population in view of previous conflicting results following comparison with both non-alcoholic and alcoholic controls. We have studied the mechanisms relevant to the influence of polymorphisms on CD14 expression by a luciferase reporter assay. Risk genotype specific expression of CD14 and other interacting proteins were studied in ALD patients and controls for understanding alcoholic disease. Overall, in this study, it has been observed that the risk and severity of alcoholic liver disease (ALD) is prevalently more intense among the ALD group, in particular among those individuals having the TT genotype of rs2569190 of the CD14 gene. The rs2569190 SNP has shown genetic association with alcoholic liver disease, and the polymorphisms may exert their effects by modulating CD14 expression.

## Data availability statement

The original contributions presented in the study are included in the article/supplementary materials. Further inquiries can be directed to the corresponding author.

## Ethics statement

The study was approved by the AII India Institute of Medical Sciences, New Delhi Institute ethics review committee with reference to IEC number IESC/T-440/26.08.15, RT-7/2015. The patients/participants provided their written informed consent to participate in this study.

## Author contributions

NR: Concept, data acquisition, draft writing. NN: Concept, data acquisition. HK: data acquisition. CP: data acquisition. JJ: data acquisition. HP: Data acquisition. PV: Statistical analysis. AS: Draft writing. YB: data acquisition, draft writing. S: study concept, draft writing. BN: Concept, data acquisition, draft writing. All authors contributed to the article and approved the submitted version.

## Funding

The DST-SERB Young Scientists fellowship (YSS/2014/000717) was awarded to Dr. Neelanjana Roy and mentor Dr. Baibaswata Nayak to carry out research at the Department of Gastroenterology, All India Institute of Medical Sciences, New Delhi, India.

## Conflict of interest

The authors declare that the research was conducted in the absence of any commercial or financial relationships that could be construed as a potential conflict of interest.

## Publisher’s note

All claims expressed in this article are solely those of the authors and do not necessarily represent those of their affiliated organizations, or those of the publisher, the editors and the reviewers. Any product that may be evaluated in this article, or claim that may be made by its manufacturer, is not guaranteed or endorsed by the publisher.
